# *Escherichia coli O157:H7* beef carcass contamination and its antibiotic resistance in Awi Zone, Northwest Ethiopia

**DOI:** 10.1002/fsn3.3550

**Published:** 2023-07-05

**Authors:** Aschalew Ayisheshim Tarekegn, Birhan Agimas Mitiku, Yeshwas Ferede Alemu

**Affiliations:** ^1^ Alage Agricultural Technical and Vocational Education and Training College Alage Ethiopia; ^2^ Department of Veterinary Science, College of Agriculture and Environmental Science Bahir Dar University Bahir Dar Ethiopia

**Keywords:** abattoir, antibiograms, Awi Zone, butcher shop, *E. coli O157:H7*

## Abstract

*Escherichia coli O157:H7* is a cause of foodborne disease and global public health issues especially in developing countries like Ethiopia. A cross‐sectional study was done from January 2022 to July 2022 in Awi Zone to assess the occurrence and antibiograms of *E. coli O157:H7*. Abattoirs and butcher shops were selected purposively, whereas a systematic random and purposive sampling technique was employed to select study units in abattoirs and butcher shops, respectively. A total of 248 swab samples were collected, isolated, and confirmed using bacteriological culture, biochemical tests, and latex agglutination tests. *Escherichia coli O1157:H7* antibiogram tests were performed using Kirby–Bauer disk diffusion method. Logistic regression was used to analyze and measure the degree of association between the presumed risk factors and *E. coli O157:H7* occurrence. The overall occurrence of *E. coli O157:H7* was estimated to be 8.87% and a relative higher (11.29%) occurrence of *E. coli O157:H7* was recorded at butcher shops when compared to abattoirs (6.45%). All isolates were susceptible to gentamicin followed by chloramphenicol (81.81%). About 81.81% of the isolates were resistant to ampicillin and 77.23% of isolates developed resistance to two and more than two antibiotics (MDR). In conclusion, *E. coli O157:H7* was detected in the study area. Thus, educating abattoir and butcher shop workers, and consumers, on hygienic handling practices and safe consumption of meat could eliminate foodborne infection associated with *E. coli O157:H7* occurrence.

## Graphical abstract

*Escherichia coli O157:H7* was carcass contaminant at selected abattoirs and butcher shops in Awi Zone. *Escherichia coli O157:H7* showed different susceptibility and resistance patterns toward antimicrobial disks. Butcher shops are the major risk factor for *E. coli O157:H7* occurrence in beef. Contamination of carcasses during transport due to open and uncleaned vehicle transportation from the abattoir to retail shops and unhygienic handling and storage of carcasses at butcher shops are the major sources of contamination.

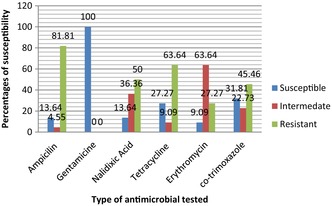

## 1 INTRODUCTION

Foodborne illness is a gastrointestinal disease mostly caused by highly infectious pathogenic microorganisms (Agueria et al., 2018). The risk of microbial foodborne disease is increasing throughout the world, even though the scientific and technological development in the livestock sector shows progressive improvement. In developing nations such as Ethiopia, they are commonly susceptible to foodborne diseases due to poor food handling and sanitation practices, insufficient food safety management, weakened monitoring systems, weakened infrastructure, and illegal slaughtering practices (Nigatu et al., 2017).

Bacterial foodborne disease is responsible for hospitalizations and deaths in the world such as *Salmonella enterica*, *Campylobacter* species, *Listeria monocytogenes*, *Staphylococcus*, *Escherichia coli* (*E. coli*), and *Enterobacter*. *Escherichia coli* are a group of bacteria that are part of the intestinal microflora of healthy animals and humans. Most of them are normal commensals found in the intestinal tract of both humans and animals, while others are pathogenic to humans. Pathogenic *E. coli* is distinguished from normal flora by their possession of virulence factors. *Escherichia coli O157:H7* is one of the known serotypes that can cause foodborne infection in humans. However, there are no regulations in Ethiopia to protect meat consumers from foodborne pathogens (Fitsum et al., 2015). In most cases, cattle are incriminated as primary reservoirs and usually asymptomatic carriers of *E. coli O157:H7*. Therefore, it is transient in the gastrointestinal tract of animals, so cattle feces can be a potential source of *E. coli O157:H7* for human infection. However, studies indicated that the main sources of this pathogen can be consumption of contaminated raw meat, raw milk, and undercooked ground beef (Ashenafi et al., 2017).

Besides the occurrence of *E. coli O157:H7*, the increasing emergence and spread of antimicrobial resistance bacteria, multidrug‐resistant zoonotic foodborne pathogens, in particular, have become a significant concern (Umaru et al., 2019). Antimicrobial resistance has been recognized as an emerging worldwide problem in human and veterinary medicine in both developed and developing countries (Nega et al., 2021) due to the wide use of antimicrobials for either therapeutic or prophylactic purposes and growth promotion in livestock. Such indiscriminate use of antimicrobials causes the development of considerable resistant bacterial strains (Mengistu & Eyob, 2020).

The antimicrobial‐resistant bacteria can be transmitted to humans through the food chain from food animal reservoirs. Studies conducted in different areas of Ethiopia indicated that there has been a significant rise in the antimicrobial resistance patterns of *E. coli O157:H7* to commonly used antimicrobials (Aklilu et al., 2022). The existence of resistant *E. coli O157:H7* isolates is a potential threat to public health. Hence, the implementation of prevention and control strategies from farm production to consumption of meat and meat products is crucial (Tizeta et al., 2014).

In Ethiopia, animals are commonly slaughtered, dressed and carcasses are distributed to consumers under unhygienic conditions that cause microbiological contamination and poor quality meat. This is due to the prevailing poor food handling and sanitation practices, inadequate food safety laws, weak regulatory systems, lack of financial resources to invest in safer equipment, and lack of training for food handlers (Nigatu et al., 2017). Persons in this country habit, choice, intension, and perception about raw beef or undercooked meat consumption is not considered a health risk. Contaminated carcass is the most common route of *E. coli O157:H7* infection in the community (Mengistu & Eyob, 2020). In Awi Zone, cattle are slaughtered under contaminated conditions at very poor municipal abattoirs and carcasses were handled and transported from the abattoir to butcher shops un‐hygienically (abattoirs and butcher shop workers' observations, unpublished data).

In Ethiopia, a number of studies have been conducted about the occurrence and antibiograms of *E. coli O157:H7* isolates from food of animal origin, animal surfaces, and samples taken from slaughterhouses, retail shops, and restaurants (Abebe et al., 2023; Aklilu et al., 2017, 2022; Ashenafi et al., 2017; Biruhtesfa et al., 2017; Eshetu et al., 2021; Habtamu et al., 2021; Melaku et al., 2013; Mengistu & Eyob, 2020; Nega et al., 2021; Rosa et al., 2017; Shimelis et al., 2017; Tizeta et al., 2014). However, most of those studies focused on the prevalence and antimicrobial susceptibility of *E. coli O157:H7*, and some of them focused on identifying the potential microbial contamination source of *E. coli O157:H7* along the chain of meat production process starting from slaughtering up to ready for human consumption and there was no previous study done on the occurrence and antibiograms of *E. coli O157:H7*, and the potential contamination points at abattoirs and butcher shops in Awi Zone. Therefore, this study was done to assess the occurrence and antibiograms of *E. coli O157:H7* from carcass, hand, knife, and hook swab samples from selected abattoirs and butcher shops in Awi Zone.

## 2 METHODS

### 2.1 Description of the study area

**FIGURE 1:**
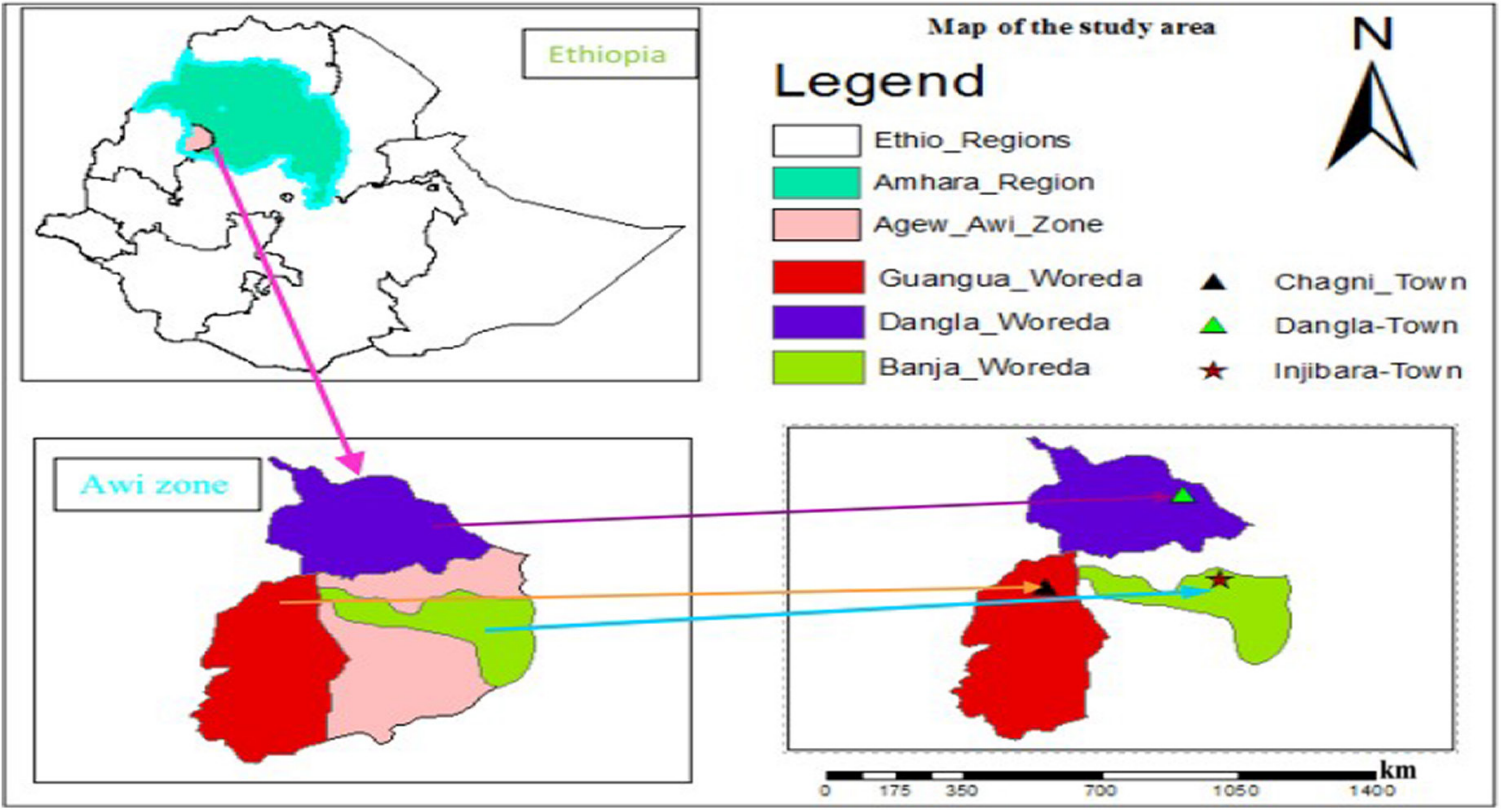
Locations of the study area (ArcMap output from Ethiopian shape file).

This study was carried out in Awi administrative Zone which is one of the three nation zones found in Amhara regional state. The Zone is divided into 15 administrative with nine rural districts and six town administrations. The study was carried out in the administrative towns of Dangla, Injibara, and Chagni (Figure 1). There was only one municipal abattoir in each town and the main source of cattle to be slaughtered at each municipal abattoir were originated from *Jawi*, *Dangla*, and *Addis Kidam* for Dangla municipal abattoir; *Injibara*, *Tilli*, and *Gimja Bet* for Injibara municipal abattoir; *Mentawuha*, *Chagni*, and *Zigem* for Chagni municipal abattoir, respectively. Swab samples were collected from cattle slaughtered at the aforementioned municipal abattoirs and the respective butcher shops received carcasses from those municipal abattoirs. The study areas are known for their high potential for livestock production and supplying meat for local consumption. According to each abattoir's information, 5–8 cattle were slaughtered per day in Dangla and Chagni municipal abattoirs, but were reached up to 10 slaughters per day in Injibara municipal abattoir. Similarly, information was obtained from each town health office; there were 37, 53, and 21 legally registered butcher shops in Dangla, Injibara, and Chagni town, respectively.

### 2.2 Study population

The source of population for swab sample collection were local zebu breeds sold for slaughter in Awi Zone towns and for those questionnaires surveyed were from abattoir and butcher shop workers in Awi Zone. The study population comprised apparently healthy cattle, which received health certificate from a nearby veterinary clinic, and ready for slaughter as well as workers who participated at the three municipal abattoirs and the respective selected butcher shops of Awi Zone. The study units were carcasses from selected local zebu cattle slaughtered and butcher men who operate the selected carcasses at abattoirs and butcher shops.

### 2.3 Study design, sample size determination, and sampling technique

A cross‐sectional study was conducted from January 2022 to July 2022 to assess the occurrence, antibiograms of *E. coli O157:H7*, and carcass contaminations at abattoirs and butcher shops in Awi Zone. The sample size was calculated using the following equation described by Thrusfield (2018).

$$\frac{n=\mathrm{Z\alpha}/2\;\;P\exp\;\left(1-P\exp\right)}{d2}$$

where *n* is the required sample size, *Zα*/2 is the *z*‐value for 95% level of confidence, *P* is the expected prevalence, and *d* is the desired absolute precision.

Using previously reported occurrence of *E. coli O157:H7* (8.9%) in Bahir Dar city, municipal abattoir (Habtamu et al., 2021), 1.96 *Zα*/2 value at a confidence level of 95%, 5% required absolute precision, the estimated sample size was 124. The sample size of butcher shop samples was equal to the respective carcass received from each municipal abattoir. Therefore, a total of 248 swabs samples (124 samples from municipal abattoirs and samples from butcher shops), were collected.

The three municipal abattoirs were selected purposively based on their functionality. Slaughter cattle were recorded during ante‐mortem inspection and lists of butcher shops were obtained from Dangila, Injibara, and Chagni town administration health office and purposive sampling techniques were used to select butcher shops. A systematic random sampling method was employed to select the study unit at each municipal abattoir and every second cattle was selected on every sampling day. Each abattoir was visited once per week on Wednesday, Monday, and Friday nights for 3 months, and an identification number was given for each animal during the ante‐mortem examination and follow‐up during postmortem examination on each day. Then, one sampling unit was selected from daily slaughtered animals and was followed them purposively to the respective butcher shops. The butcher shop workers' hand swab samples, knife, and hook were collected based on the role they had in manipulation and processing of the selected cattle carcasses. The samples from butcher shops were collected from those who received this carcass.

### 2.4 Data collection

The swab samples for the study were collected under strict aseptic procedures. Selected swab sample was swabbed using the method described in ISO17604, (2015) by placing a sterile template (10 × 10 cm) on the surface of the abdomen, thorax, and brisket, which are sites with the highest rate of contamination for carcass swabbing and (5 × 5 cm) limited areas on the surface of hands, knives, and hooks from abattoirs and butcher shops. A sterile cotton‐tipped swab (2 × 3 cm) fitted with a shaft, was first soaked in 10 mL of buffered peptone water (Himedia), and rubbed first horizontally and then vertically at least five times on the surface of carcasses, hands, knives, and hooks. On completion of the rubbing process, the shaft was broken in the inner wall of screw cupped universal bottle containing 10 mL of buffered peptone water and disposed leaving the cotton swab in the bottle. All collected samples were properly labeled with sample code, sample type, date of collection, and sources of samples. Then, the samples were transported in an ice box containing ice packs in a cold chain to Bahir Dar University Institute of Biotechnology laboratory for microbiological analysis.

### 2.5 Isolation and identification of *E. coli O157:H7*

Upon arrival at Bahir Dar University Institute of Biotechnology laboratory, each sample was streaked on MacConkey agar (Himedia), which was a selective and differential medium of *E. coli* and incubated aerobically at 37°C for 24 h for primary isolation. The plates were observed for the growth of *E. coli*. Then, a single, isolated colony of typical bright pink on MacConkey agar was subcultured on Eosin Methylene Blue agar (EMB) (Himedia). Moreover, a single colony black with green metallic sheen character on EMB, which was the typical feature of *E. coli* was subcultured to 1% sorbitol MacConkey Bases with cefixime and tellurite supplements (CT‐ SMAC), a selective agar medium for *E. coli O157:H7* isolation. Nonsorbitol fermenters' pale colonies on CT‐SMAC were suspected as *E. coli O157:H7*. Then, the suspected colonies were transferred to Nutrient agar for biochemical tests.

Presumptive *E. coli O157:H7* isolates were characterized for their biochemical activity using indole, methyl red, Vogues Proskaeur, and citrate utilization (IMViC) tests as well as triple sugar iron (TSI) test. Isolates from sorbitol MacConkey agar and identified (+ + − −) IMViC patterns for indole, methyl red, Vogues Proskaeur, and citrate utilization tests, respectively, as well as triple sugar iron, yellow butt and slant, and gas production, were considered as *E. coli O157:H7*. The latex agglutination test was intended for confirmatory identification of *E. coli* serogroups *O157* and *H7* antigens. The NSF isolates from CT‐ SMAC were inoculated to nutrient agar for refreshment of colonies. A drop of test latex (*O157* and *H7* coated latex in different circles of the reaction card) and 20 μL of sterile saline water were dispensed into the reaction card separately. Then, up to three suspected colonies to be tested were emulsified using a sterile single plastic loop in a drop of sterile saline solution on the test circle. After ensuring a smooth suspension of bacterial colonies and saline, the test latex was mixed with the emulsified test sample. The contents of the circle were then mixed carefully by spreading the latex over the entire area of the circle. Finally, the card has rocked in a circular motion for 1 min and examined for agglutinations. Colonies and the test latex giving a precipitation reaction within 1 min were confirmed as positive results (Aklilu et al., 2022). Negative results were reported if no agglutination occurred and a smooth suspension remained after 1 min in the test area. Therefore, an isolate was considered as *E. coli O157:H7* if and only if agglutination occurred in both *O157* and *H7* coated latex tests.

### 2.6 Antibiograms of *E. coli O157:H7*

Phenotypic antimicrobial susceptibility tests were performed using the Kirby–Bauer Disk Diffusion Susceptibility Test Protocol on Mueller Hinton agar (OXOID) (Hudzicki, 2009). The criteria used to select the antimicrobials disks were based on the regular use of antimicrobials in ruminants, availability, and from CLSI suggested guidelines for antimicrobial susceptibility testing of *Enterobacteriaceae* (CLSI, 2020). Thus, antimicrobial disks used in this study were ampicillin, gentamicin, erythromycin, tetracycline, chloramphenicol, trimethoprim/sulfamethoxazole, and nalidixic acid (Himedia) (Table 1).

**TABLE 1:** Antimicrobial disks used for antibiograms of *Escherichia coli O157:H7*.

Antimicrobial agent	Disk content (μg)	Interpretive categories and zone diameter breakpoints, nearest whole mm		
		S (≥)	I	R (≤)
Ampicillin	10	17	14–16	13
Gentamicin	10	15	13–14	12
Nalidixic acid	30	19	14–18	13
Tetracycline	30	15	12–14	11
Erythromycin	15	23	14–22	13
Chloramphenicol	30	18	13–17	12
Trimethoprim/Sulfamethoxazole	25	16	11–15	10

Abbreviations: I, Intermediate; R, Resistant; S, Susceptible.

Up to three 24 h, incubated fresh colonies of *E. coli O157:H7* were taken from nutrient agar using a sterile inoculating loop, suspended in a test tube with 2 mL of sterile saline and the saline tube was vortexed to create a smooth suspension. The turbidity of the suspension was adjusted at 0.5 McFarland standard by adding more organisms when the suspension was too light, or diluted with sterile saline when the suspension was too heavy in which McFarland Densitometer was used as a standard measurement for checking the turbidity.

Sterile cotton swab was dipped into the suspension, rotated several times, pressed firmly on the inside wall of the tube above the fluid level to remove excess inoculums, and was swabbed uniformly over the surface of Muller Hinton agar plate within a sterile safety cabinet. The plates were held at room temperature for 15 min to allow drying. Then, antimicrobial disks with known concentrations of antimicrobials were placed on a cultured Muller Hinton agar plate at an appropriate distance with flamed forceps and incubated for 24 h at 37°C. Following incubation, the diameter of the zone of inhibition was measured by using a ruler to the nearest millimeter. Interpretations of the results were depending on the categorization of isolates into susceptible, intermediate, or resistant (CLSI, 2020).

### 2.7 Data management and statistical analysis

The data were classified, arranged, filtered, coded, and entered into Microsoft Office Excel spreadsheet 2021. Following entry, the data were exported to STATA version 16 for appropriate statistical analysis. Descriptive statistics were performed to look the data pattern and frequency distribution of various risk factors was applied to quantify the occurrence of carcass contamination and antibiograms of the isolates. Logistic regression analysis was employed to measure the degree of association between the presumed risk factors and *E. coli O157:H7* occurrence. The degree of association between individual risk factors and outcome variables was screened by univariable logistic regression analysis using 25% or *p* < .25. Those variables significantly associated with the outcome variable using univariable logistic regression analysis were recruited for multivariable logistic regression analysis to see their independent effect. In multivariable logistic regression analysis, a model was fitted for each outcome variable by stepwise backward elimination of insignificant variables (*p* > .05). Multivariable logistic regression was used to see statistically significant association among risk factors. The data were interpreted as significant when the *p* value was less than .05.

### 2.8 Ethical clearance

The study was conducted after the protocol was ethically reviewed and approved by the Institutional Review Board of Bahir Dar University, College of Agriculture and Environmental Sciences. Therefore, before the study run, ethical clearance letters (certificate Ref. No: 1/93/1.3.4., Date: 12/04/2014, E.C.) were received from this board. Letters of support were obtained from Bahir Dar University and official permission was requested from Dangla, Injibara, and Chagni town service office higher officials to abattoir administrators and health office.

## 3 RESULTS

In this study, a total of 248 swab samples were taken from the municipal abattoirs and butcher shops. Then, the swabs were subjected to bacteriological tests and bacterial isolates from the swabs were subjected to biochemical tests. After a series of bacteriological and biochemical tests, 29 isolates were suspected as *E. coli* and confirmation by latex agglutination tests indicated that 22 (75.86%) of them were found to be *E. coli O157:H7*. Therefore, the current study revealed that the overall occurrence of *E. coli O157:H7* was 22/248 (8.87%). Of these, eight isolates, 8/124 (6.45%) were from municipal abattoirs and 14 isolates, 14/124 (11.29%) were from butcher shops. Accordingly, from abattoir samples, *E. coli O157:H7* were detected in 5/31 (16.13%) of carcass, 1/31 (3.23%) of hand swabs, 1/31 (3.23%) of knife swabs, and 1/31 (3.23%) of hook swab samples. Among the examined abattoir swab samples, the highest (16.13%) and the lowest (3.23%) occurrences of *E. coli O157:H7* were recorded from carcass swab and the rest swab samples, respectively. The difference in the occurrence of *E. coli O157:H7* among different abattoir swab sample types were not statistically significant (*p* > .05). Similarly, from the butcher shop samples, *E. coli O157:H7* was detected in 8/31 (25.80%) of carcass, 1/31 (3.23%) of hand, 2/31 (6.45%) of knife, and 3/31 (9.68%) of hook swab samples. Among the examined butcher shop swab sample types, the highest (25.80%) and lowest (3.23%) occurrences of *E. coli O157:H7* were recorded from carcass swab and hand swab samples, respectively. There was also statistically no difference in the occurrence of *E. coli O157:H7* among different butcher shop swab sample types (*p* > .05) (Table 2).

**TABLE 2:** Occurrence of *Escherichia coli O157:H7* isolates from different abattoirs and butcher shop swab sample types in Awi Zone, Northwest Ethiopia.

Sample source	Sample type	No. of samples tested	No. of positive (%)	*p*‐value
Abattoir	Carcass	31	5 (16.13)	.157
	Hand	31	1 (3.23)	
	Knife	31	1 (3.23)	
	Hook	31	1 (3.23)	
Subtotal		124	8 (6.45)	
Butcher shops	Carcass	31	8 (25.80)	.083
	Hand	31	1 (3.23)	
	Knife	31	2 (6.45)	
	Hook	31	3 (9.68)	
Subtotal		124	14 (11.29)	
Grand total		248	22 (8.87)	

The study area, sample source, and sample types were considered as possible risk factors. Univariable logistic regression analysis was performed to understand the association of each predictor variable with the occurrence of *E. coli O157:H7*. A statistically significant difference was observed (*p* < .05) in the study area (Dangla), sample source, and sample type (carcass). The occurrence of *E. coli O157:H7* recorded from carcass swab samples 13 (21%) was significantly higher than knife 3 (4.84%) and hook (6.45%) swab samples. Similarly, the occurrence of *E. coli O157:H7* recorded from butcher shop swab samples 14 (11.29%) was significantly higher than in abattoir 8 (6.45%) swab samples and the occurrence recorded in Dangla 13 (16.25%) was significantly higher than Injibara sourced swab samples (Table 3).

**TABLE 3:** Risk factors affecting the occurrence of *Escherichia coli O157:H7* using univariable logistic regression analysis in Awi Zone, Northwest Ethiopia.

Risk factors	No. of examined	No of positive (%)	OR (95% CI)	*p*‐value
Sample type				
Hand	62	2 (3.23)	Ref.	
Carcass	62	13 (21)	3.85 (1.18–12.56)	.03*
Knife	62	3 (4.84)	0.74 (0.16–3.44)	.69
Hook	62	4 (6.45)	1.53 (0.25–9.46)	.55
Sample source				
Abattoir	124	8 (6.45)	Ref.	
Butcher Shop	124	14 (11.29)	2.55 (1.03–6.32)	.04*
Study area				
Chagni	80	4 (5)	Ref.	
Dangla	80	13 (16.25)	3.58 (1.12–11.52)	.03*
Injibara	88	5 (5.68)	1.13 (0.29–4.37)	.86

Abbreviations: CI, Confidence interval; OR, odds ratio; Ref., Reference.

* Significant.

Except some estimated statistical parameter variations, all those significant risk factors screened by univariable logistic regression were also found significant factors affecting the occurrences of *E. coli O157:H7* in multivariable logistic regression analysis. The odds of detection of *E. coli O157:H7* occurrence was 4.08 times more likely to occur in carcass swab samples than in hand swab samples. Likewise, the occurrence of *E. coli O157:H7* recorded from butcher shop swab samples (11.29%) was significantly higher than from abattoir (6.45%) and the odds of detection of *E. coli O157:H7* occurrence was 2.67 times more likely to occur in butcher shops than abattoirs. Moreover, the occurrence of *E. coli O157:H7* recorded in Dangla town swab samples (16.25%) was significantly higher than in Injibara town (5.68%), showing that the odds of detection of *E. coli O157:H7* occurrence was four times more likely to occur in Dangla town than in Chagni town (Table 4).

**TABLE 4:** Risk factors affecting the occurrences of *Escherichia coli O157:H7* using multivariable logistic regression analysis in Awi Zone, Northwest Ethiopia.

Risk factors	No. of examined	No of positive (%)	AOR (95% CI)	*p*‐value
Sample type				
Hand	62	2 (3.23)	Ref.	
Carcass	62	13 (21)	4.08 (1.20–13.91)	.04*
Knife	62	3 (4.84)	0.73 (0.15–3.50)	.69
Hook	62	4 (6.45)	1.14 (0.12–2.53)	.51
Sample source				
Abattoir	124	8 (6.45)	Ref.	
Butcher shop	124	14 (11.29)	2.67 (1.02–7.00)	.05*
Study area				
Chagni	80	4 (5)	Ref.	
Dangla	80	13 (16.25)	4.00 (1.12–13.57)	.04*
Injibara	88	5 (5.68)	1.28 (0.31–5.17)	.86

Abbreviations: AOR, adjusted odds ratio; CI, Confidence interval; Ref., Reference.

* Significant.

### 3.1 Antibiograms of *E. coli O157:H7*

**FIGURE 2:**
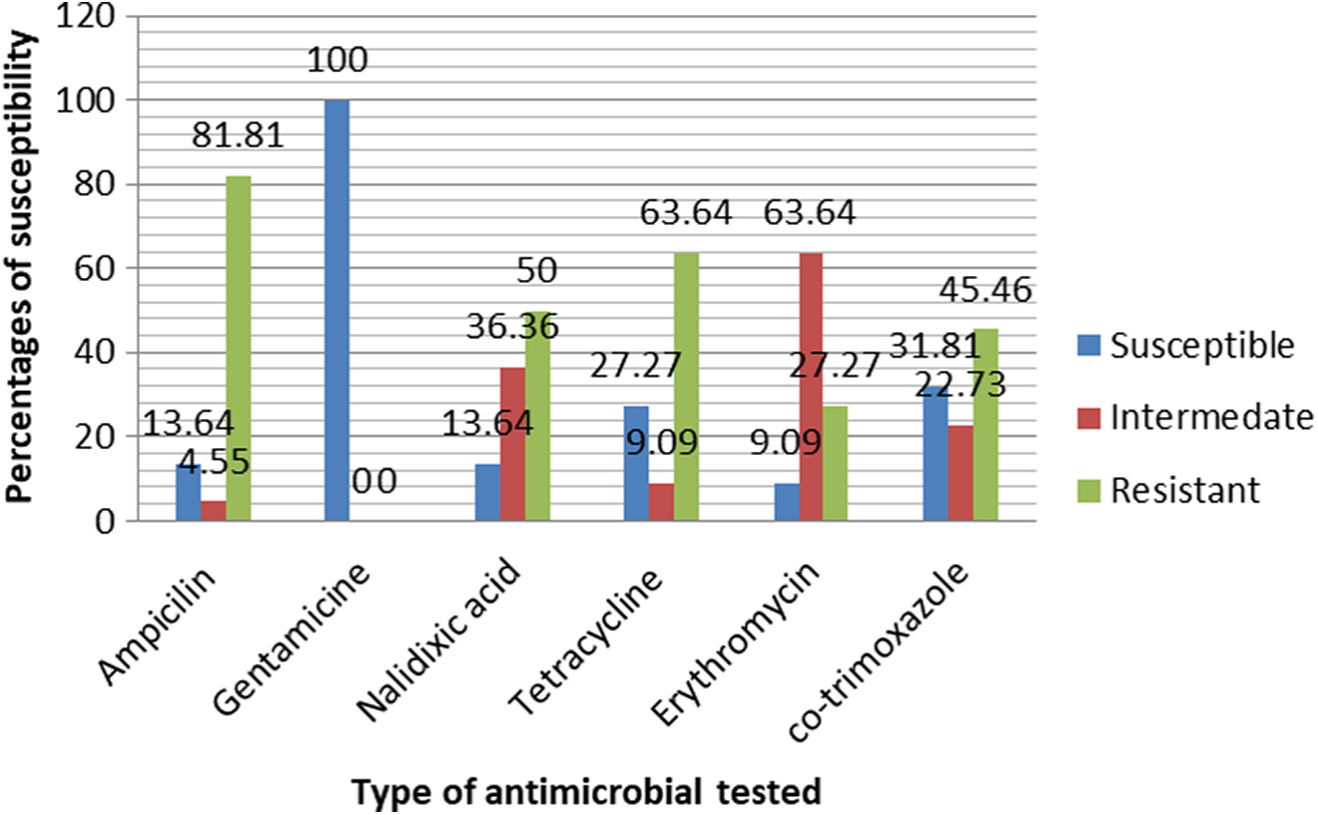
Percentage‐wise antibiograms of *Escherichia coli O157:H7* isolates with antimicrobial agents.

In this study, *E. coli O157:H7* showed different susceptibility and resistance patterns toward seven antimicrobial disks. Out of the seven antimicrobials tested for susceptibility pattern, only two (9.09%) isolates were susceptible to all antimicrobial disks. Moreover, 22 (100%) isolates were susceptible to gentamicin. On the other hand, 18 (81.81%) isolates were resistant to ampicillin followed by tetracycline 14 (63.64%) and nalidixic acid (50%). In addition, 14 (63.64%) isolates showed intermediate susceptibility patterns to erythromycin (Figure 2).

#### 3.1.1 Monodrug‐resistant profile of *E. coli O157:H7*

**FIGURE 3:**
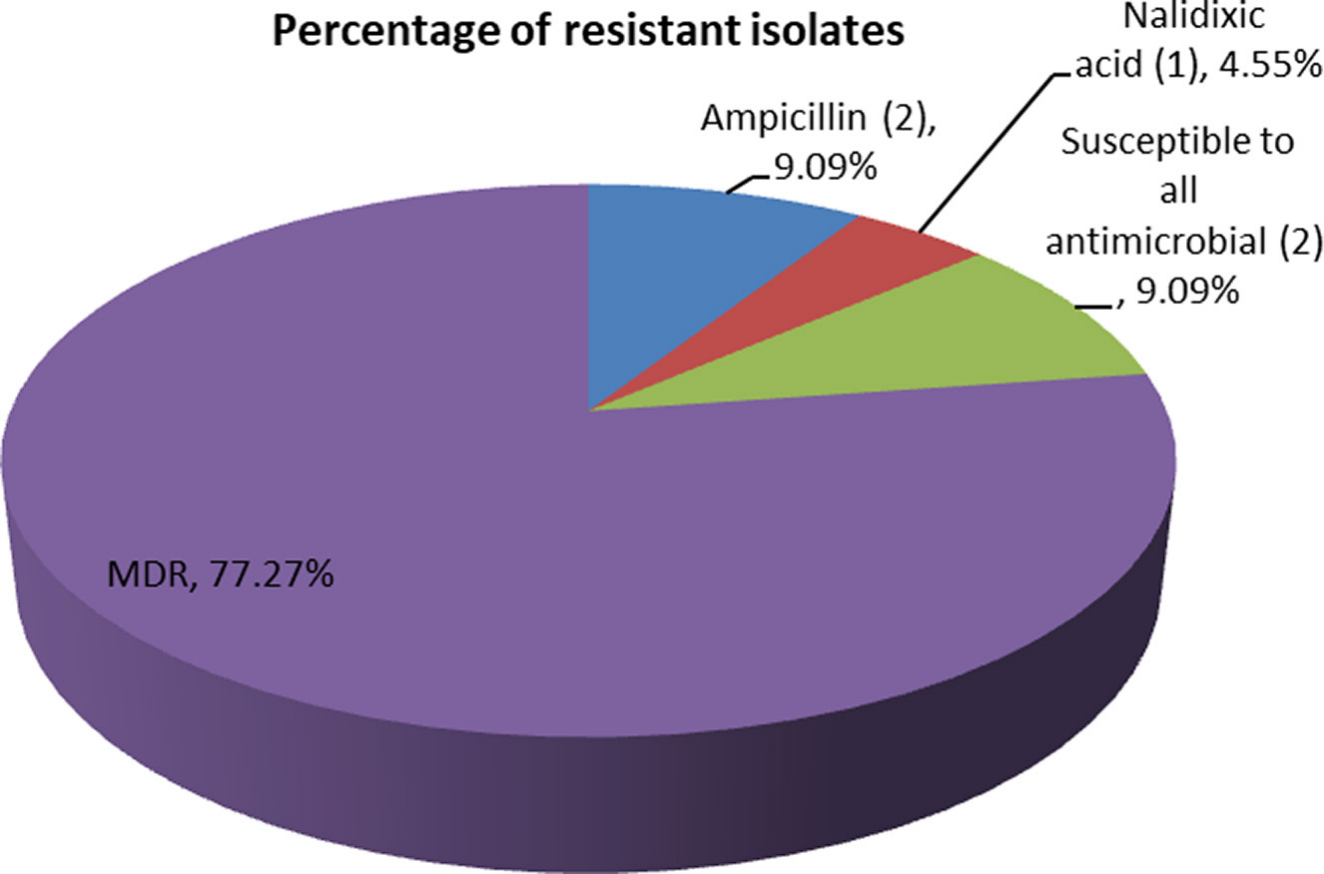
Monodrug resistance of *Escherichia coli O157:H7* isolates to antimicrobial agents.

In this study, from all *E. coli O157:H7* isolates, 20 (90.90%) of them showed single to five drug resistances and only 3 (13.64%) of them showed monodrug resistance (Figure 3).

#### 3.1.2 Multidrug‐resistant profile of *E. coli O157:H7*

For our purposes here, we chose to define multidrug resistance as the resistance of a single isolate to two and more than two drugs. Of 22 *E. coli O157:H7* isolates, 17 (77.27%) of them were showed MDR (Figure 3). Two isolates were resistant to two antimicrobial agents and four isolates were resistant to three antimicrobial disks, likewise, four and seven isolates were resistant to four and five antimicrobial agents, respectively. The most frequent resistance combination was observed at 31.81% in five antimicrobial agent combinations of ampicillin, nalidixic acid, tetracycline, erythromycin, and trimethoprim/sulfamethoxazole (Table 5).

**TABLE 5:** Multidrug‐resistant patterns of *Escherichia coli O157:H7* isolates.

Number of antimicrobial resistances	Frequency (%) of resistant isolates	Type of antimicrobial agents
Two	1 (4.55%)	AM + ERY
	1 (4.55%)	AM + COT
Three	4 (18.18%)	AM + ERY
		AM + Te + ERY
		AM + NA + COT
		NA + Te + ERY
Four	4 (18.18%)	AM + Te + ERY + COT
		AM + NA + Te + ERY (2)
		AM + NA + Te + COT
Five	7 (31.81%)	AM + Te + ERY + COT (2)
		AM + NA + Te + ERY + TS (5)

Abbreviations: AM, ampicillin; ERY, erythromycin; NA, nalidixic acid; Te, tetracycline; TS, trimethoprim/sulfamethoxazole.

## 4 DISCUSSION

### 4.1 Occurrence of *E. coli O157:H7*

The present study revealed that the overall occurrence of *E. coli O157:H7* in Awi Zone was 8.87%. This result was found comparable with other reports with 5.4% in Jimma town (Mengistu & Eyob, 2020) and 9.1% in Ambo Town (Nega et al., 2021) from Ethiopia. Additionally, comparable results were also reported in other parts of the world, such as 6.5% in USA (Jamie et al., 2014), 13.3% in China (Shuhong et al., 2015), and 7.86% in Iran (Zohreh, 2018). However, the present finding was higher than some other scholars reported, such as 2.65% in Haramaya University (Melaku et al., 2013), 1.3% in Addis Ababa (Rosa et al., 2017), 2.4% in Hawassa (Biruhtesfa et al., 2017), 4.9% in Mojo (Solomon et al., 2019), and 1.39% in Jimma town (Eshetu et al., 2021). Additionally, lower findings were also reported from other countries such as 2.3% in Egypt (Ahmed et al., 2017), and 0.2% in Nigeria (Mailafia et al., 2017). On the other hand, other scholars reported higher results than the present findings such as 13.3% in Addis Ababa (Tizeta et al., 2014) from Ethiopia. In Africa, 35.1% of occurrences were reported in South Africa (Onyeka et al., 2021). Moreover, reported in the world were included 21.23% in Iran (Momtaz & Jamshidi, 2013), 22% (Vinothkumar et al., 2014) and 25.46% (Vijayan et al., 2017) in India, and 60% in Nigeria (Lennox et al., 2020). Overall variations in the occurrence of *E. coli O157:H7* might be due to the difference in sample size, sampling techniques, and study area difference.

The current finding also revealed that the occurrence of *E. coli O157:H7* was 6.45% at the abattoir level, lower than 11.29% at butcher shop levels. This finding is in line with other scholars reported in Ethiopia such as 4.7% and 21.9% prevalence in abattoirs and retail shops, respectively, in Addis Ababa (Tizeta et al., 2014), 0.54% and 0.8% prevalence from processing plants carcass internal swab samples and retail shops carcass swab, respectively, in Addis Ababa (Rosa et al., 2017), as well as 7.2% and 19.2% from abattoir and retail shops, respectively, in Ambo town (Nega et al., 2021). This similar finding and the higher prevalence in retail shops may be as a result of increased contamination of carcasses during transport due to open and uncleaned vehicle transportation from the abattoir to retail shops and unhygienic handling and storage of carcasses at butcher shops. In addition to this, meat could get further contamination in butcher shops during preparation by personal and equipment contacts. On the other hand, this finding disagrees with the occurrence of *E. coli O157:H7* from abattoir was higher than butcher shop level such as 6.0% and 4.5% occurrence from abattoir house and butcher shops, respectively, in Jimma town (Mengistu & Eyob, 2020), 2.77% and 1.38% from abattoir carcass swabs and butcher shop meat, respectively, isolated from cattle meat at Jimma (Eshetu et al., 2021), and 2.7% and 2% from abattoir carcass swab and butcher shops meat sample, respectively, in Hawassa (Biruhtesfa et al., 2017). These slight differences might be due to the difference in faulty sampling techniques and laboratory procedures.

The finding in the current study shows that 6.45% of abattoir was comparable with the reported 7.3% in Jimma (Aklilu et al., 2017) and 6.3% in Bishoftu (Fanta et al., 2020) from Ethiopia and 7.2% from abattoir carcass swab samples in Jordan (Osaili et al., 2013). This similarity could be due to the similar sample size, sampling technique, and laboratory techniques employed. This occurrence was higher than in studies in other countries that reported 0.3% in European Union (EFSA & ECDC, 2013) and 2.2% in Nigeria (Tafida et al., 2014). The difference in the occurrence of *E. coli O157:H7* in beef can be due to differences in the hygienic handling practices during slaughter and handling of beef carcasses. In similar conditions, the occurrence of 11.29% from butcher shops in the present study was higher than 3.3%, 4%, and 4% of raw beef samples from butcher shops in Holeta and Batu, respectively (Ashenafi et al., 2017), 1% in Dire Dawa retail shops (Shimelis et al., 2017), 1.38% from butcher shops in Jimma city (Eshetu et al., 2021), and 3.64% from retail raw beef in Addis Ababa (Aklilu et al., 2022) from Ethiopia and 6.4% in Iran (Rahimi et al., 2012). On the other hand, this finding was lower than 23.5% reported from retail raw meat in Iran (Panahee & Pourtaghi, 2017). The variation of these findings might be the difference in sanitation condition of abattoirs and butcher shops.

In this study, 21% of carcass swabs were positive for *E. coli O157:H7*. This result was higher than 10.4% in selected woredas of Tigray (Abebe & Tafese, 2014), 4.5% in butcher shops and restaurants in central Ethiopia (Ashenafi et al., 2017), and 3.07% in Bishoftu town (Segni et al., 2018) from Ethiopia. Additionally, lower results from other countries, 2.8% in Iran (Zarei et al., 2013), 2.86% in China (Jacob et al., 2014), 2% from raw beef carcass samples in Riyadh, Saudi Arabia (Hessain et al., 2015), 13.32% in retail fresh raw meat in South China (Shuhong et al., 2015), 7.41% carcass samples in Malaysia (Kwan et al., 2019), and 10.5% from meat products sold in Obinze abattoir, IMO state, Nigeria (Osazee & Shadrach, 2020). On the other hand, this finding is lower than 25.5% reported from ground beef samples in Argentina (Victoria et al., 2013). These variations could be due to differences in the hygienic conditions of meat preparation and processing. Moreover, the presence of pathogens on workers' hands occurred as a result of contact with cattle fecal matter during slaughter processing (Tan et al., 2013). The present finding revealed that 3.23% of *E. coli O157:H7* were isolated from workers' hand. This finding disagreement reported by other researchers shows 9.7% of abattoir workers' hand during working activities in halal cattle abattoirs in Peninsular Malaysia (Shamsul et al., 2016) and higher than 0% at butcher shops and restaurants in central Ethiopia (Ashenafi et al., 2017).

Likewise, the occurrence of *E. coli O157:H7* from the knife swab sample, 4.84% in the current study, consistent with 5% reported from Mojo export abattoir (Solomon et al., 2019) from Ethiopia and 3.3% reported from knife swab samples in Argentina (Victoria et al., 2013). This result was lower than 9.3% from Jimma municipal abattoir (Aklilu et al., 2017), and 12% from carcass swabs at Addis Ababa municipal abattoir (Muhammed et al., 2018). On the other hand, this finding was higher than 0% at butcher shops and restaurants in central Ethiopia (Ashenafi et al., 2017).

The overall variations in the prevalence of *E. coli O157:H7* might be due to different sampling techniques, laboratory methodology used, agroecology of the study area, and hygienic conditions used.

Moreover, this study revealed that the occurrence of *E. coli O157:H7* in hook swab samples was 6.45%. This finding was much lower than with 50% of Haremaya University slaughterhouse equipment's swab samples (Shimelis et al., 2017). On the other hand, this finding was higher than 4.16% of abattoir meat hanging up a wire swab in Jimma, Ethiopia (Eshetu et al., 2021). This finding difference may be due to the latex agglutination identification technique of this study and the biology identification of the others, which was a highly sensitive automated machine.

### 4.2 Antibiograms of *E. coli O157:H7* isolates

Antimicrobial resistance has become a global concern. Indiscriminate use of antimicrobial agents in humans and veterinary medicine is considered as the most important factor for promoting the emergence, selection, and dissemination of resistant microorganisms in both veterinary and human medicine (Mude et al., 2017). In this study, the antibiograms of *E. coli O157:H7* isolates to the seven antimicrobials were tested and the antibiograms of the isolates were graded according to CLSI (2020).

In this study, all (100%) and 81.81% of the isolates were susceptible to gentamycin and chloramphenicol, respectively. This finding was aligned with reported 100% susceptibility to gentamicin (Biruhtesfa et al., 2017; Muhammed et al., 2018; Tizeta et al., 2014). But this outcome was contradicted with the finding of 83.3% resistance to chloramphenicol in Jimma town (Mengistu & Eyob, 2020) and 65.9% to gentamicin in Ambo town (Nega et al., 2021). On the other side, the current study revealed that 81.81%, 63.64%, and 50% of the isolates were resistant to ampicillin, tetracycline, and nalidixic acid, respectively. The ampicillin resistance in this finding was similar agreement with reports in Somali Region of Ethiopia (Fitsum et al., 2015) and Bishoftu town (Segni et al., 2018). Moreover, the finding of tetracycline and nalidixic acid resistance in this study was in line with the report from Haramaya University (Melaku et al., 2013). This was attributed to the repeated misuse of these antimicrobials by their animals for prophylaxis and/or treatment purposes. However, this finding disagrees with 100% susceptibility to tetracycline and nalidixic acid from slaughterhouses and beef carcasses at retail shops in Ethiopia (Rosa et al., 2017) and only 50% of the isolates had resistance to ampicillin from beef and buffalo samples in India (Kwan et al., 2019).

Multidrug resistance has been a common problem among gram‐negative bacterial species (Tizeta et al., 2014). Concurrent resistance of *E. coli O157:H7* to some antimicrobials may complicate the therapeutic management of infection. In the present study, multidrug resistance was observed to five antimicrobials including ampicillin, nalidixic acid, tetracycline, erythromycin, and trimethoprim/sulfamethoxazole. Of the aforementioned antimicrobials, resistance to ampicillin was observed in most isolates. This result was in agreement with findings from different other studies in Ethiopia (Aklilu et al., 2022; Mengistu & Eyob, 2020). Unlike previously 92.8% sensitivity to nalidixic acid was reported, in the current study, only 50% of the isolates were resistant to nalidixic acid (Aklilu et al., 2022). The possible reason for the difference in the degree of susceptibility and resistance may be the result of temporal and geographical differences between the current and previous studies. And that 74.21% of respondents using appropriate gloves reduced carcass contamination in Nigeria (Gbolabo et al., 2020).

## 5 CONCLUSION AND RECOMMENDATIONS

This study revealed that *E. coli O157:H7* was carcass contaminant at selected abattoirs and butcher shops in Awi Zone. The majority of carcasses delivered to Awi Zone butcher shops were handled in unhygienic conditions at ambient temperatures, with inadequate sanitation in carcass transporting vehicles. The occurrence of *E. coli O157:H7* reported in butcher shops was higher than in abattoirs. Occurrences of *E. coli O157:H7* from carcass swab samples were higher than hand, knife, and hook swab samples. The study also confirmed that all *E. coli O157:H7* isolates were susceptible to gentamycin and most of them were susceptible to chloramphenicol. However, near to all the isolates were resistant to single to five antibiotics and most of the isolates showed MDR. The highest resistance was observed to ampicillin followed by tetracycline and nalidixic acid. Generally, this study could give an insight into the occurrence of *E. coli O157:H7* and its public health significance associated with the consumption of unsafe meat. Therefore, carcass swab samples were considered as a significant source of *E. coli O157:H7* foodborne diseases and spreading of antimicrobial resistance to the consumers in the study area. Based on the above gaps and conclusions, the following recommendations are forwarded:

1. In the abattoir and butcher shops, standard operating measures must be practiced.
2. Slaughterhouse administrators and butcher shop owners should collaborate with meat inspection groups to deliver and provide safe beef to consumers.
3. Veterinarians and healthcare professionals should collaborate to create public awareness about the serious health risks involved with the consumption of raw meat.

## AUTHOR CONTRIBUTIONS

**Aschalew Ayisheshim Tarekegn:** Conceptualization (equal); data curation (equal); formal analysis (equal); funding acquisition (equal); investigation (equal); methodology (equal); software (equal); validation (equal); writing – original draft (equal). **Birhan Agimas Mitiku:** Conceptualization (equal); data curation (equal); formal analysis (equal); investigation (equal); methodology (equal); project administration (equal); resources (equal); software (equal); supervision (equal); validation (equal); visualization (equal); writing – original draft (equal); writing – review and editing (equal). **Yeshwas Ferede Alemu:** Conceptualization (equal); data curation (equal); formal analysis (equal); investigation (equal); methodology (equal); project administration (equal); resources (equal); software (equal); supervision (equal); validation (equal); visualization (equal); writing – review and editing (equal).

## FUNDING INFORMATION

This work was supported by Alage Agricultural Technical and Vocational Education and Training College, Ministry of Labor and Skills, Ethiopia; ref. No. 334/02/02.

## CONFLICT OF INTEREST STATEMENT

The authors declare that they have no competing interests.

## Data Availability

All information is available upon reasonable request from the corresponding author.
